# HSP90AA1 Facilitates Vascular Calcification in Chronic Kidney Disease Involving Chaperone-Mediated Autophagy

**DOI:** 10.3390/biomedicines14040881

**Published:** 2026-04-12

**Authors:** Yaling Zhang, Ming Li, Yanwen Luo, Liming Huang, Sipei Chen, Guisen Li, Yi Li, Li Wang

**Affiliations:** 1 Department of Nephrology and Institute, Sichuan Provincial People’s Hospital, School of Medicine, University of Electronic Science and Technology of China, Chengdu 610056, China; zhangyalingcd@med.uestc.edu.cn (Y.Z.); yanwenxluo@uestc.edu.cn (Y.L.); liminghuang1@163.com (L.H.); 15671620476@163.com (S.C.); guisenli@163.com (G.L.); liyisn@med.uestc.edu.cn (Y.L.); 2 Department of Cardiovascular, Sichuan Provincial People’s Hospital, University of Electronic Science and Technology of China, Chengdu 610056, China; lmjy4788@126.com

**Keywords:** chronic kidney disease, vascular calcification, vascular smooth muscle cells, chaperone-mediated autophagy, LAMP2a, HSP90AA1

## Abstract

**Background:** Chronic kidney disease (CKD) associated vascular calcification (VC) is a leading cause of cardiovascular mortality, partially driven by osteogenic transdifferentiation of vascular smooth muscle cells (VSMCs). Chaperone-mediated autophagy (CMA) is a selective lysosomal degradation cellular process. However, the precise role and mechanism of CMA in CKD-associated vascular calcification remain unknown. **Methods:** We studied calcified arteries from CKD patients and rats fed on a high-phosphate diet using histological and ultrastructural methods. VSMCs’ calcification was induced by a calcification medium containing high phosphate and calcium. CMA activity was measured by a KFERQ reporter and lysosomal staining. The expression of LAMP2a and HSP90AA1 was knocked down by siRNA, overexpressed by plasmid, and activated by QX77.1. Bioinformatic analysis, protein interaction studies, immunofluorescence and co-immunoprecipitation were performed to investigate the potential mechanism of CMA in VC. **Results:** The expression of LAMP2a was increased in human calcified radial artery tissues (*n* = 3, *p* < 0.05) and rats’ calcified aortic tissues (*n* = 3, *p* < 0.01), accompanied by lysosomal abnormalities. The activity of CMA was increased during the osteogenic transdifferentiation of VSMCs, as indicated by increased expression of RUNX2 and reduced expression of SM22α (*p* < 0.05). LAMP2a knockdown attenuated VSMCs’ calcification (*p* < 0.05), whereas pharmacological activation of CMA aggravated calcification in VSMCs (*p* < 0.01). Bioinformatic screening identified HSP90AA1 as a candidate involved in CMA in vascular calcification. Elevated HSP90AA1 expression was observed in human calcified radial artery tissues (*n* = 3, *p* < 0.01) and rat calcified aortic tissues (*n* = 3, *p* < 0.01), which promoted osteogenic transdifferentiation of VSMCs (*p* < 0.05). HSP90AA1 interacted with LAMP2a and positively regulated its expression (*p* < 0.01). **Conclusions:** These findings support an association between CMA activation and CKD vascular calcification. It suggests that HSP90AA1 facilitates vascular calcification in chronic kidney disease involving chaperone-mediated autophagy.

## 1. Introduction

Chronic kidney disease (CKD) has become a significant global public health concern, with its prevalence continuously rising [1]. Vascular calcification (VC) is recognized as one of its most serious and potentially fatal complications [2,3]. VC is characterized by pathological deposition of calcium–phosphate crystals within the arterial wall, particularly in the medial layer, leading to arterial stiffening, reduced vascular compliance, and an elevated risk of cardiovascular events [3,4]. Notably, patients with end-stage renal disease (ESRD) receiving maintenance hemodialysis (MHD) exhibit a remarkably high prevalence of VC, which constitutes a primary factor contributing to mortality in this population [5,6]. Nevertheless, the fundamental mechanisms driving vascular calcification in ESRD remain not fully understood, and effective strategies for its prevention or treatment are currently unavailable.

Vascular smooth muscle cells (VSMCs) play a central role in the pathogenesis of VC. Under CKD-associated pathological conditions, such as hyperphosphatemia, hypercalcemia, and accumulation of uremic toxins, VSMCs undergo osteogenic transdifferentiation, in which they lose their contractile phenotype and acquire osteoblast-like characteristics [7,8]. This phenotypic transition is accompanied by upregulation of osteogenic markers (e.g., RUNX2) and downregulation of smooth muscle-specific markers (e.g., SM22α), ultimately facilitating calcium deposition in the vascular wall [9,10]. Accordingly, elucidating the regulatory networks governing VSMC osteogenic transdifferentiation is crucial for understanding the development and progression of CKD-related VC [11,12].

Chaperone-mediated autophagy (CMA), one of the three core autophagic pathways, is a lysosome-dependent process that mediates the highly selective degradation of soluble cytosolic proteins [13,14]. In contrast to macroautophagy and microautophagy, chaperone-mediated autophagy (CMA) operates independently of vesicular membrane dynamics. Instead, it relies on the coordinated function of molecular chaperones and lysosomal receptors to mediate precise substrate recognition and subsequent transmembrane degradation. Through this unique mechanism, CMA serves as a critical pathway for maintaining cellular proteostasis and regulating fundamental physiological processes [15,16]. CMA has been implicated in the pathogenesis of a broad spectrum of disorders, including neurodegenerative diseases, cardiovascular and cerebrovascular diseases, lipid metabolic disorders, and malignancies [17,18]. Its functional dysregulation induces pathological protein misfolding and aggregation, endothelial cell senescence, glucose and lipid metabolic disturbances, oxidative stress [19,20], and other aberrant cellular processes. However, the precise role of CMA in CKD vascular calcification is still unclear.

This study aimed to investigate the role of CMA in CKD vascular calcification, as well as its underlying mechanisms. We examined LAMP2a expression and lysosomal changes in calcified arterial tissues from MHD patients and CKD rats, assessed CMA activity and VSMC osteogenic transdifferentiation under high calcium/phosphate conditions. This study could explore the regulatory mechanisms of CMA in CKD vascular calcification and could find potential therapeutic targets for CKD vascular calcification.

## 2. Materials and Methods

### 2.1. Study Subjects and Animal Models

#### 2.1.1. Human Tissue Samples

Calcified radial artery tissues were obtained from discarded radial artery tissues of maintenance hemodialysis patients who underwent forearm arteriovenous fistula construction, whereas non-CKD control arterial tissues were obtained from patients undergoing limb amputation for orthopedic trauma who had no prior underlying diseases associated with vascular lesions. The inclusion criteria (all must be satisfied): Age ≥ 18 years; history of CKD ≥ 3 months and scheduled for forearm arteriovenous fistula surgery; evidence of arterial calcification; voluntary participation in the study. Exclusion Criteria (any single criterion): Age < 18 years; pregnancy or lactation; history of tumor, neurodegenerative disease, liver cirrhosis, acute inflammation, or immune dysfunction; no evidence of arterial calcification; unwillingness to participate in the study. In the present exploratory tissue study, three samples per group were available for histological comparison, consistent with the quantitative analyses shown in the figures. All participants provided written informed consent, and the human study was approved by the Ethics Committee of Sichuan Provincial People’s Hospital (approval no. 2021.233). Tissue specimens were either immediately fixed in 4% paraformaldehyde for subsequent histological staining.

#### 2.1.2. Animal Models

Male Wistar rats (200–250 g) aged 8–10 weeks were obtained from DOSSY (Chengdu, China), and were fed and watered ad libitum, and housed under specific pathogen-free conditions at Sichuan Provincial People’s Hospital. All procedures were approved by the Institutional Ethics Committee of Sichuan Provincial People’s Hospital (approval no. 2021.233).

The CKD-associated vascular calcification rat model was established as described in our previous study [21]. Following randomization, fourteen rats were initially allocated to each experimental group. The Pi + 5/6 Nx rat model was established via 5/6 nephrectomy combined with feeding a high-phosphorus diet containing 1.2% phosphorus (Pi). The 5/6 nephrectomy was performed using a two-stage surgical procedure: rats were anesthetized by intraperitoneal injection of sodium pentobarbital at a dosage of 40 mg/kg, after which the left renal pedicle was ligated, and the entire left kidney was excised. One week after the initial surgery, the upper and lower poles of the right kidney were ligated to resect approximately two-thirds of the right renal tissue. Following completion of the 5/6 nephrectomy, rats in the Pi + 5/6 Nx group were fed the aforementioned high-phosphorus diet for 16 consecutive weeks. Rats in the sham group underwent sham surgical procedures without renal tissue resection and were maintained on a standard normal diet. Five rats survived in both the model group and the sham operation group; at 16 weeks, all surviving rats were euthanized for the collection of peripheral blood samples and aortic tissue specimens.

### 2.2. Cell Culture and Treatment

Rat thoracic aortic VSMCs (A7r5, China) were obtained from the National Collection of Authenticated Cell Cultures. The cells were maintained in Dulbecco’s modified Eagle’s medium (DMEM) (Gibco, 10566016, Waltham, MA, USA) supplemented with 10% fetal bovine serum (FBS) (Gibco, 10099-141c, USA) and 1% penicillin–streptomycin (Hyclone, SV30010, Logan, UT, USA) at 37 °C in a humidified incubator with 5% CO_2_.

To induce osteogenic transdifferentiation and calcification, VSMCs were cultured in calcification medium containing 10 mM β-glycerophosphate (Sigma, G9422, Burbank, CA, USA) and 1.5 mM calcium chloride (Solarbio, C7250, Beijing, China) for five days [22,23] (Supplementary Figure S1). For pharmacological activation of CMA, cells were treated with 10 μM QX77.1 (Selleck, Houston, TX, USA) during the calcification period. Control cells (CTRL) were cultured in standard DMEM without additional supplements.

### 2.3. Cell Transfections

#### 2.3.1. Plasmid Transfection

The PAmCherry1-KFERQ-NEtag plasmid, pRP-EGFP/Hygro-EF1A-rHsp90aa1 plasmid (Hsp90aa1 plasmid) or pRP-EGFP/Hygro-EF1A plasmid (control plasmid) (VectorBuilder, Guangzhou, China) was transfected into VSMCs using Lipofectamine 3000 (L3000015, Thermo Fisher Scientific, Waltham, MA, USA).

VSMCs were seeded into 6-well plates. When the cell confluence reached 60%, the medium in each well was replaced with serum-free and antibiotic-free DMEM, and cells were incubated for 30 min to adapt. Transfection systems were prepared using the corresponding plasmids as required, adding 2 μg plasmid to 125 μL serum-free DMEM and incubating for 5 min at room temperature. The activated Lipofectamine 3000 dilution was gently mixed with the plasmid dilution and incubated for 15–20 min at room temperature to form stable complexes. The plasmid complexes were then added dropwise to each well, gently rocked for uniform distribution, and incubated statically at 37 °C with 5% CO_2_ for 6 h. Overexpression efficiency was assessed by Western blotting. Independent transfection experiments were repeated at least three times.

#### 2.3.2. Small Interfering RNA (siRNA) Transfection

siRNAs targeting rat LAMP2a (siLAMP2a), HSP90AA1 (siHSP90AA1), and non-targeting control siRNA (siNC) were purchased from RIBOBIO (Guangzhou, China). VSMCs were cultured to 60% confluence, and 50 nM siRNA was diluted in serum-free DMEM, while riboFECT CP (RIBOBIO, Guangzhou, China) was diluted in serum-free DMEM. The two dilutions were gently mixed and incubated at room temperature for 10 min to form complexes, which were then added to the cells for static incubation at 37 °C with 5% CO_2_ for 48 h. Western blot analysis was performed to evaluate gene silencing efficiency, and each experiment was performed in at least three independent biological replicates.

### 2.4. Alizarin Red S Staining

Tissue sections (5 μm thickness) or cultured VSMCs were fixed in 4% paraformaldehyde (Bioshark, Guangzhou, China) for 30 min, rinsed with phosphate-buffered saline (PBS), and stained with 2% Alizarin Red S solution (pH 4.2) for 10 min at room temperature. Excess dye was removed by washing with distilled water, and calcium deposits were visualized as red staining under a light microscope. All staining results were quantitatively scored using the H-score method via the IHC Profiler plugin of ImageJ software 1.53a (National Institutes of Health, USA), with the calculation formula: H-Score = (1 × % weak positive) + (2 × % moderate positive) + (3 × % strong positive) (weak/moderate/strong positive refers to stained areas for Alizarin Red staining and stained cells for IHC staining). All experiments were performed with three biological replicates (*n* = 3) per group, and all quantification processes were conducted in a single-blinded manner (evaluators unaware of sample grouping) to avoid subjective bias [24].

For Alizarin Red S staining, VSMCs were fixed with 4% paraformaldehyde for 30 min. Following thorough washing, the cells were stained with Alizarin Red S solution (ALIR-10001, Cyagen Biosciences, Suzhou, China) for 10 min. Subsequently, images were captured using a digital camera.

### 2.5. Von Kossa Staining

Von Kossa staining was performed on paraffin-embedded tissue sections. Sections were fixed with 4% paraformaldehyde, air-dried, and exposed to a 5% silver nitrate solution under ultraviolet light for 30 min. Following rinsing with distilled water, sections were treated with 5% sodium thiosulfate for 5 min and counterstained with hematoxylin. Calcified regions appeared as black deposits under microscopic examination. All staining results were quantitatively scored using the H-score method via the IHC Profiler plugin of ImageJ 1.53a software (National Institutes of Health, USA), with the calculation formula: H-Score = (1 × % weak positive) + (2 × % moderate positive) + (3 × % strong positive) (weak/moderate/strong positive refers to stained areas for Alizarin Red staining and stained cells for IHC staining). All experiments were performed with three biological replicates (*n* = 3) per group, and all quantification processes were conducted in a single-blinded manner (evaluators unaware of sample grouping) to avoid subjective bias [24].

### 2.6. Hematoxylin-Eosin (HE) Staining

Tissue sections were dewaxed, rehydrated through graded ethanol, and stained with hematoxylin for 5 min, followed by differentiation in 1% hydrochloric acid–ethanol. Sections were then counterstained with eosin for 3 min, dehydrated, cleared, and mounted. Histological structures were examined under a light microscope.

### 2.7. Immunohistochemistry (IHC)

Deparaffinized tissue sections underwent antigen retrieval by boiling in citrate buffer (pH 6.0) for 15 min. Endogenous peroxidase activity was blocked with 3% hydrogen peroxide for 10 min. Sections were incubated overnight at 4 °C with primary antibodies against LAMP2a (1:200, #ab125068, Abcam, Cambridge, UK) or HSP90AA1 (1:1000, #13171-1-AP, Proteintech, Rosemont, IL, USA), followed by incubation with horseradish peroxidase-conjugated secondary antibody (#PV-9000, ZSGB-BIO, Beijing, China) for 1 h at room temperature. Immunoreactivity was visualized using 3,3′-diaminobenzidine (DAB) substrate, and nuclei were counterstained with hematoxylin. Positive staining appeared brown, and images were acquired using a light microscope. Semi-quantitative evaluation was performed in ImageJ using representative fields from each biological replicate.

### 2.8. Immunofluorescence Staining

Deparaffinized tissue sections and cultured VSMCs were fixed with 4% paraformaldehyde, permeabilized with 0.3% Triton X-100 for 15 min, and blocked with 5% bovine serum albumin (BSA) (Bioshark, China) for 1 h. Sections/cells were then incubated with primary antibodies against LAMP2a (1:100, #ab125068, Cambridge, UK) and HSP90AA1 (1:200, #13171-1-AP, Proteintech, USA) overnight at 4 °C. Following PBS washes, samples were incubated with fluorescein isothiocyanate (FITC)-conjugated secondary antibody (1:300, #511201, Zenbio, Shanghai, China) and Cy5-conjugated secondary antibody (1:300, #550083, Zenbio, China) for 1 h at room temperature in the dark. Nuclei were counterstained with 4′,6-diamidino-2-phenylindole (DAPI) (ANT063, AntGene, Cambridge, MA, USA) for 5 min. Images were acquired using a laser scanning confocal microscope (LSM980, Zeiss, Oberkochen, Germany).

VSMCs transfected with the PAmCherry1-KFERQ plasmid were incubated with 50 nM LysoTracker Green (#L7526, Thermo, USA) for 30 min at 37 °C to label lysosomes. stained with DAPI (ANT063, AntGene), and examined under a confocal microscope (LSM980, Zeiss, Germany) to assess co-localization of PAmCherry1-KFERQ and LysoTracker Green.

### 2.9. Western Blot (WB) Analysis

Total protein was extracted from VSMCs using RIPA lysis buffer containing protease and phosphatase inhibitors (Beyotime, Haimen, China). Protein concentrations were measured with a BCA protein assay kit (Biosharp, Suzhou, China). Equal amounts of protein (30 μg per lane) were resolved on 10% SDS-PAGE gels and transferred onto PVDF membranes. Membranes were blocked with 5% non-fat milk for 1 h at room temperature and incubated overnight at 4 °C with primary antibodies against LAMP2a (1:2000, #ab125068, Abcam), HSP90AA1 (1:2000, #13171-1-AP, Proteintech, USA), Runx2 (1:2000, #ET1612-47, Huabio, Hangzhou, China), SM22α (1:2000, #10493-1-AP, Proteintech, USA), and β-actin (1:5000, #HRP-60008, Proteintech, USA), followed by HRP-conjugated secondary antibody (1:5000, #RGAR001, Proteintech, USA) for 1 h at room temperature. Protein bands were detected using an enhanced chemiluminescence system (OI600, China). Densitometric analysis was performed in ImageJ, normalized to β-actin, and summarized from at least three independent biological replicates. GraphPad Prism 10.1.2 software was used for graphing.

### 2.10. Transmission Electron Microscopy (TEM)

Arterial tissue samples were cut into approximately 1 mm^3^ pieces, fixed in 2.5% glutaraldehyde at 4 °C for 4 h, and post-fixed in 1% osmium tetroxide for 1.5 h. Samples were dehydrated through a graded ethanol series, embedded in epoxy resin, and sectioned into ultrathin sections (approximately 70 nm). Sections were stained with uranyl acetate and lead citrate and examined using a transmission electron microscope (JEOL JEM-1400, Akishima City, Japan) to analyze lysosomal morphology.

### 2.11. Bioinformatics Analysis

The GSE146638 dataset was obtained from the Gene Expression Omnibus (GEO) database. Differential gene expression analysis between uremic and control samples was conducted using the R software (version 4.1.3, R Foundation for Statistical Computing, Austria) with the limma package (version 3.60.4), with differentially expressed genes defined by|log_2_ fold change (logFC)| > 1 and *p* < 0.05. To generate a broad candidate set for CMA-related genes, we combined GeneCards [25,26] searches using the keyword “chaperone-mediated autophagy’’, removed duplicates, and used the resulting list for exploratory intersection analysis with vascular calcification-related differentially expressed genes. Protein–protein interaction (PPI) analysis was then used to prioritize candidate hub genes for subsequent experimental validation.

### 2.12. Co-Immunoprecipitation (Co-IP) Assay

The interaction between LAMP2a and HSP90AA1 in VSMCs was investigated using the Pierce Crosslink Immunoprecipitation Kit (#26147, Thermo Scientific, USA). Immunoprecipitation was performed with anti-LAMP2a (1:60, #ab125068, Abcam, UK) and anti-HSP90AA1 (1:60, #13171-1-AP, Proteintech, USA) antibodies. Finally, the membranes were imaged using a fully automated chemiluminescent gel documentation system (OI600; China). Protein band densities were quantified using ImageJ software.

### 2.13. Statistical Analysis

All experiments were independently repeated at least three times unless otherwise indicated. Data are expressed as mean ± standard deviation (SD). Statistical analyses were performed using GraphPad Prism 10.1.2. Comparisons between two groups were conducted using unpaired Student’s *t*-tests, and comparisons among multiple groups were conducted using one-way ANOVA followed by appropriate post hoc testing. Given the small exploratory sample sizes in the tissue studies, findings from those analyses should be interpreted cautiously. A *p*-value < 0.05 was considered statistically significant.

## 3. Results

### 3.1. LAMP2a Upregulation and Lysosomal Alterations in CKD-Associated Vascular Calcification

A total of six radial artery tissues and two ulnar artery tissues were collected. Of these, three vascular tissues were pathologically confirmed to exhibit calcification by Alizarin Red S staining and Von Kossa staining (mean age of 63.7 ± 27.7 years, 2 males and 1 female). Vascular specimens were collected from discarded vascular tissues of these patients. Laboratory parameters, including serum creatinine (934.6 ± 157.4 μmol/L), serum calcium (2.19 ± 0.09 mmol/L), and serum phosphate (1.76 ± 0.52 mmol/L), were recorded. The control group included three patients undergoing limb amputation due to orthopedic trauma without vascular calcification (mean age 55 ± 1.4 years, two males and one female).

**Figure 1:**
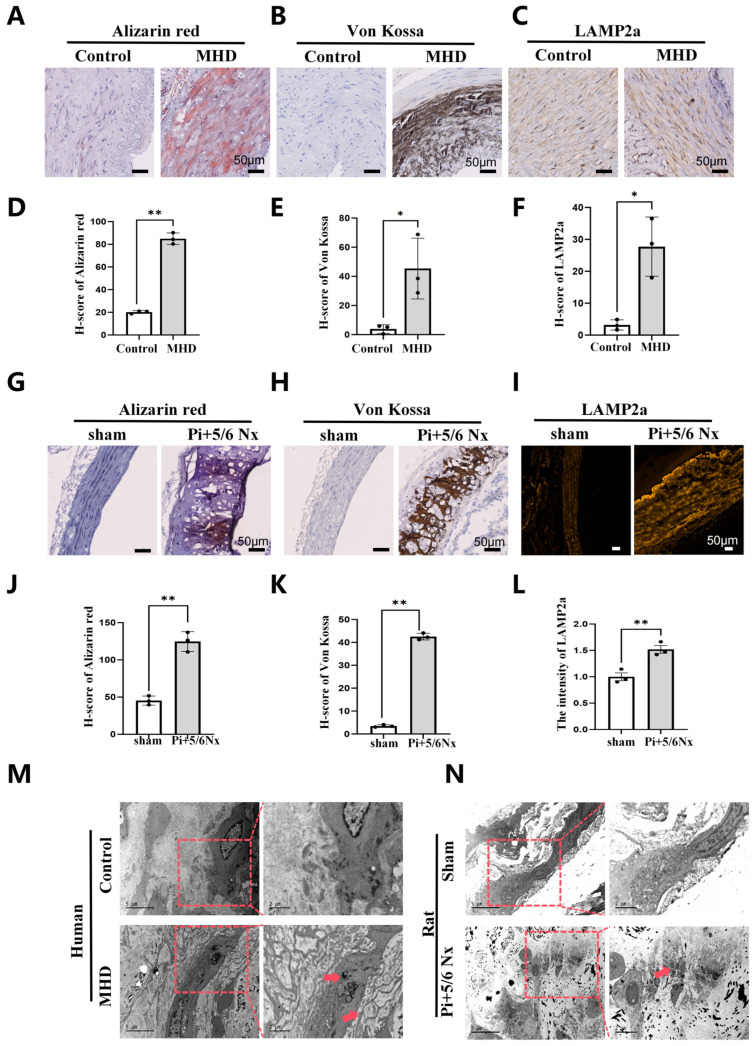
LAMP2a upregulation and lysosomal alterations in CKD-associated vascular calcification. (**A**–**F**) Alizarin Red S, Von Kossa, and LAMP2a staining of radial arteries from control and maintenance hemodialysis (MHD) patients, with corresponding quantification. (**G**–**L**) Alizarin Red S, Von Kossa, and LAMP2a staining of abdominal aortas from sham and Pi + 5/6 Nx rats, with corresponding quantification. (**M**,**N**) Transmission electron micrographs of lysosomal morphology in arteries from CKD patients (**M**) and Pi + 5/6 Nx rats (**N**). Red arrows highlight lysosomes with electron-dense contents. Mean ± SD, *n* = 3 per group.* *p* < 0.05, ** *p* < 0.01.

Alizarin Red S and Von Kossa staining revealed prominent calcium deposition in the tunica media of radial arteries from CKD patients compared with control subjects. Concurrently, LAMP2a expression was significantly upregulated in calcified radial arterial tissues (Figure 1A–F). The abdominal aortas of Pi + 5/6 Nx rat models displayed marked calcium phosphate deposition, accompanied by higher LAMP2a expression compared with the sham-operated group (Figure 1G–L). Transmission electron microscopy further demonstrated profound alterations in lysosomal morphology under vascular calcification (VC) conditions, typified by increased lysosomal abundance and enlarged size, with electron-dense degradative substrates visible within a subset of lysosomes (Figure 1M,N). Collectively, these observations indicate that autophagic activity—particularly lysosome-associated processes—may be potentiated in the pathological setting of CKD-associated VC.

### 3.2. Increased CMA Activity Coincides with Osteogenic Transdifferentiation in Calcified VSMCs

**Figure 2:**
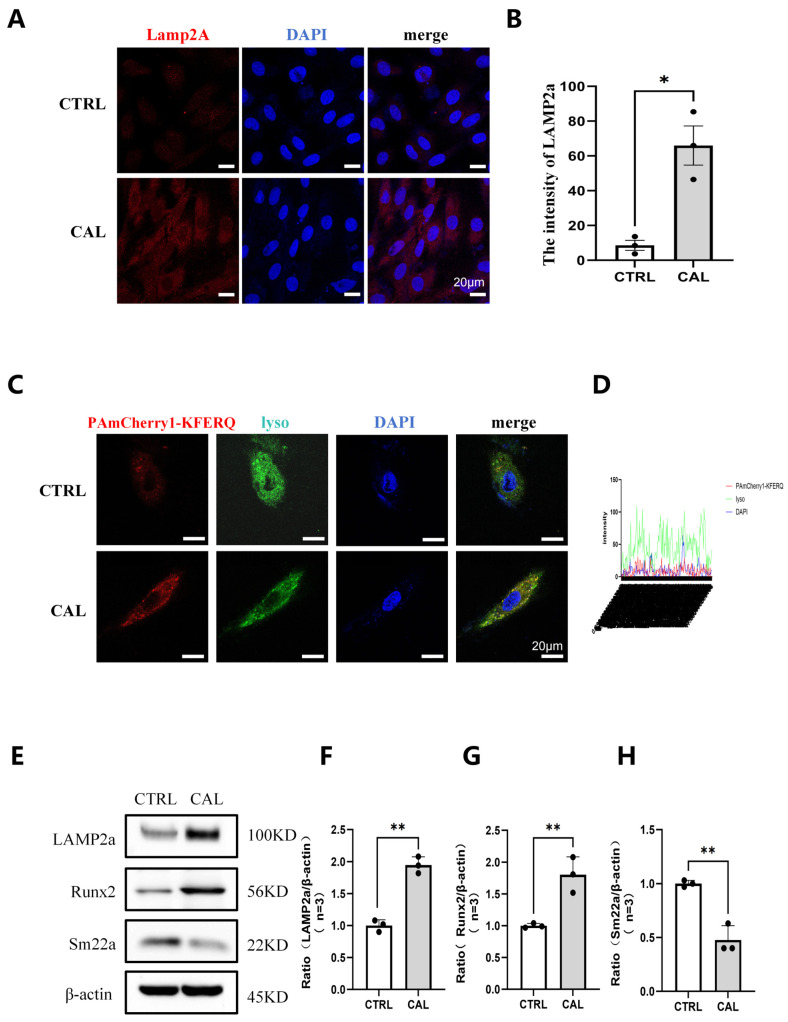
Increased CMA activity coincides with osteogenic transdifferentiation in calcified VSMCs. (**A**,**B**) Immunofluorescence staining (**A**) and quantification (**B**) of LAMP2a expression in control (CTRL) and calcified (CAL) VSMCs. Scale bar: 20 μm. (**C**,**D**) Representative images (**C**) and intensity plot (**D**) showing co-localization of PAmCherry1-KFERQ (red) and LysoTracker (green) in CTRL and CAL VSMCs. Scale bar: 10 μm. (**E**–**H**) Western blot analysis (**E**) and quantification (**F**–**H**) of LAMP2a, Runx2, and SM22α expression in CTRL and CAL VSMCs. β-actin was used as a loading control. Data are mean ± SD (*n* = 3). * *p* < 0.05, ** *p* < 0.01.

Osteogenic transdifferentiation of VSMCs was successfully induced by culturing cells in calcification medium supplemented with 10 mM β-glycerophosphate and 1.5 mM calcium chloride. Compared with VSMCs in the control group, calcified VSMCs exhibited higher expression levels of LAMP2a (Figure 2A,B). We constructed the PAmCherry1-KFERQ plasmid containing the KFERQ motif and a red fluorescent tag (Figure 2B) and transfected it into VSMCs, while lysosomes were labeled with LysoTracker. Under high calcium and high phosphate conditions, a greater proportion of PAmCherry1-KFERQ signal co-localized with cytoplasmic lysosomes, indicating increased CMA substrate translocation (Figure 2C,D). Each point on the plot corresponds to the fluorescence intensity of the two markers at the same position along the region of interest, reflecting the overlap of the two signals. When the intensity peaks of the two channels coincide, it indicates co-localization of the CMA substrate and lysosomes. In addition, VSMCs exposed to high calcium and high phosphorus exhibited elevated RUNX2 and reduced SM22α expression, accompanied by increased LAMP2a expression (Figure 2E–H). Taken together, these findings suggest that CMA is activated and may participate in the osteogenic transdifferentiation of VSMCs under high calcium and high phosphate conditions.

### 3.3. CMA Drives High Calcium/Phosphorus-Induced VSMC Osteogenic Transdifferentiation

**Figure 3:**
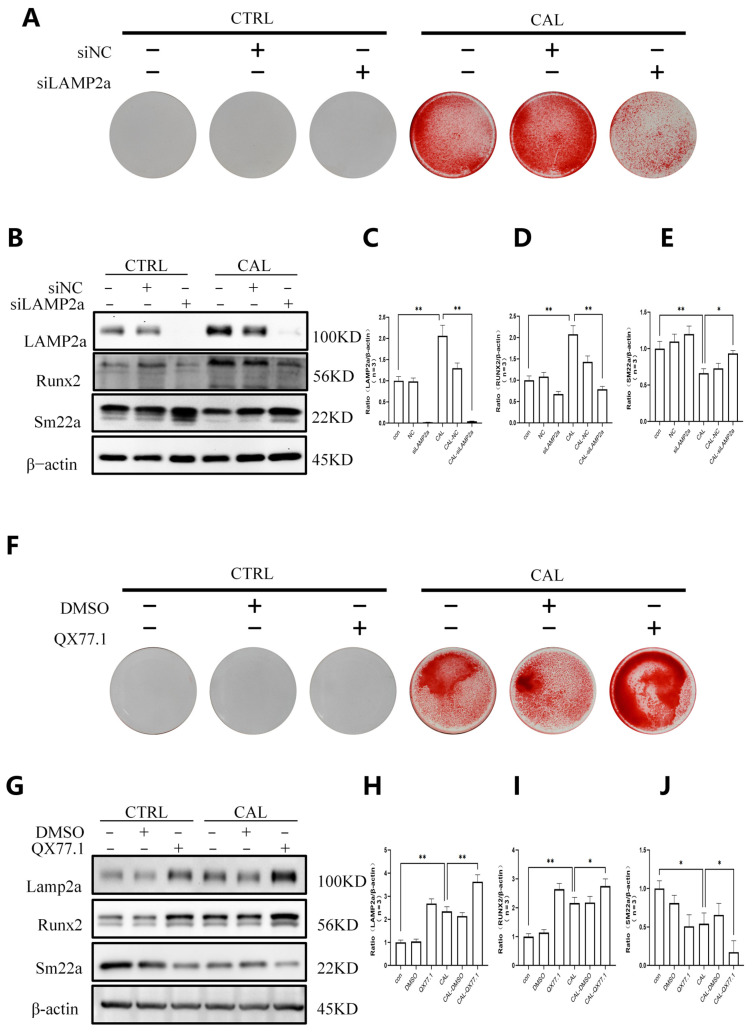
CMA drives high calcium/phosphate-induced VSMC osteogenic transdifferentiation. (**A**–**E**) Alizarin Red S staining (**A**) and immunoblot analysis (**B**–**E**) of control (CTRL) and calcified (CAL) VSMCs transfected with siNC or siLAMP2a. LAMP2a knockdown reduces calcium deposition and reverses Runx2 upregulation and SM22α downregulation in CAL VSMCs. (**F**–**J**) Alizarin Red S staining (**F**) and immunoblot analysis (**G**–**J**) of CTRL and CAL VSMCs treated with DMSO or QX77.1 (CMA agonist). QX77.1 exacerbates calcium deposition and enhances Runx2 upregulation and SM22α downregulation in CAL VSMCs. β-actin was used as a loading control. Data are mean ± SD (*n* = 3). * *p* < 0.05, ** *p* < 0.01.

To investigate the functional role of LAMP2a in VC, we silenced LAMP2a expression in VSMCs using siRNA. Alizarin Red S staining showed that LAMP2a knockdown attenuated calcium deposition in VSMCs cultured under high calcium/phosphate conditions (Figure 3A). Western blot analysis further showed that LAMP2a protein expression was elevated in calcified VSMCs, together with increased Runx2 and decreased SM22α expression, and that these changes were partially reversed by siLAMP2a (Figure 3B–E).

To further modulate CMA, we treated cells with the CMA agonist QX77.1. Alizarin Red S staining demonstrated that QX77.1 treatment aggravated calcium deposition in VSMCs (Figure 3F). Consistently, Western blot analysis showed that QX77.1 further enhanced LAMP2a expression in calcified VSMCs and promoted upregulation of Runx2 and downregulation of SM22α (Figure 3G–J). Collectively, these results indicate that under high calcium and phosphate conditions, downregulation of LAMP2a attenuates calcium deposition and osteogenic transdifferentiation of VSMCs, whereas upregulation of LAMP2a promotes VSMC calcification and osteogenic phenotypic transformation by enhancing CMA activity, highlighting a critical regulatory role of LAMP2a in VC progression.

### 3.4. HSP90AA1 Is a Key Mediator of CMA During CKD-Associated Vascular Calcification

**Figure 4:**
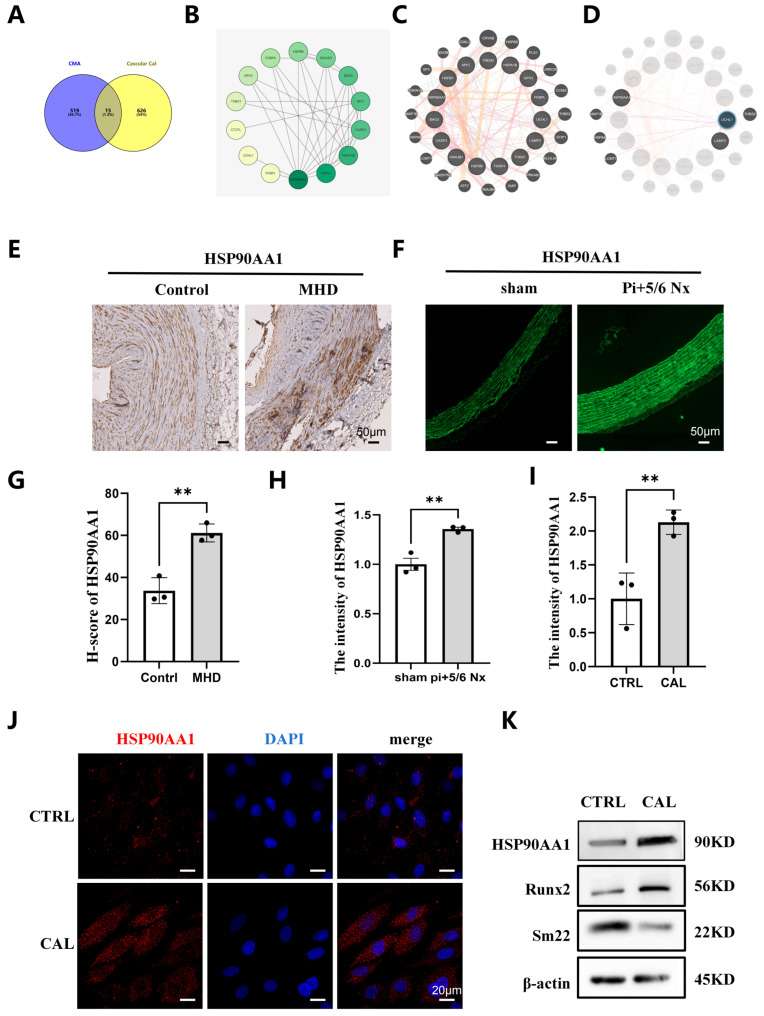
HSP90AA1 expression was significantly increased in vascular calcification. (**A**–**D**) Bioinformatics analysis identifies HSP90AA1 as a core regulator linking CMA and VC. Venn diagram (**A**) shows 15 overlapping targets between CMA-related genes and VC-associated DEGs from GSE146638. PPI network (**B**–**D**) reveals HSP90AA1 as a hub node, predicted to interact with LAMP2 via UCHL1. (**E**–**H**) IHC (**E**,**G**) and immunofluorescence (**F**,**H**) show HSP90AA1 upregulation in radial arteries from MHD patients (**E**,**G**) and abdominal aortas from Pi + 5/6 Nx rats (**F**,**H**), compared with controls. Scale bars: 50 μm. (**I**–**K**) Immunofluorescence (**I**,**J**) and immunoblot (**K**) confirm HSP90AA1 upregulation in calcified (CAL) VSMCs, alongside Runx2 upregulation and SM22α downregulation, relative to control (CTRL) cells. β-actin was used as a loading control. Data are mean ± SD. ** *p* < 0.01.

To identify candidate mediators of CMA involving VC, we analyzed the GSE146638 dataset from the GEO database. Differential gene expression analysis between uremic and control samples was conducted using the R package “limma,” with DEGs defined as those with |log fold change (logFC)| > 1 and *p* value < 0.05. A total of 534 CMA-related genes were retrieved from the GENECARD and OMIM databases. Intersection analysis between these CMA-related genes and VC-associated genes yielded 15 overlapping targets (Figure 4A). Among these, HSP90AA1 exhibited the strongest interaction with other genes in the PPI network and was predicted to interact with LAMP2 via UCHL1 (Figure 4B,D).

To validate the bioinformatic predictions, we performed IHC staining on calcified radial artery tissues from MHD patients. IHC and immunofluorescence analyses showed elevated HSP90AA1 expression in calcified arteries from MHD patients and abdominal aortas of Pi + 5/6 Nx rats relative to controls (Figure 4E–H). Consistently, immunofluorescence staining and Western blot analysis confirmed HSP90AA1 upregulation in VSMCs cultured under high calcium/phosphate conditions (Figure 4I–K).

### 3.5. HSP90AA1 Promotes VSMC Osteogenic Transdifferentiation and Calcification

**Figure 5:**
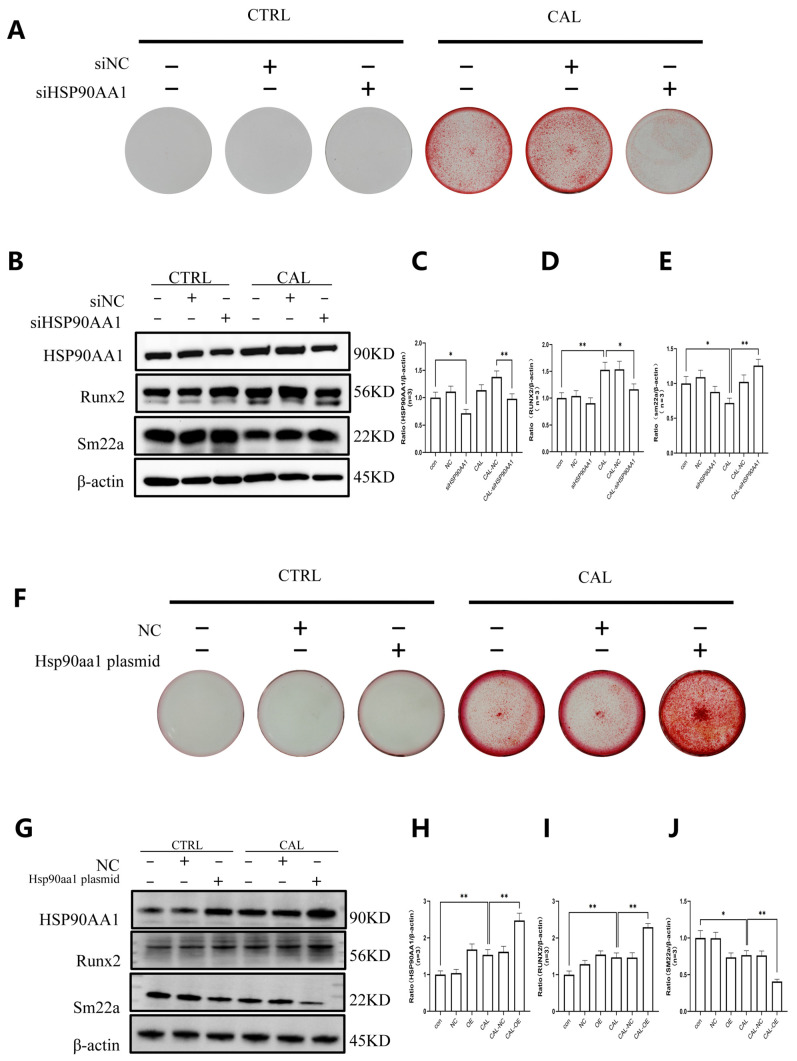
HSP90AA1 promotes VSMC osteogenic transdifferentiation and calcification. (**A**–**E**) Alizarin Red S staining (**A**) and immunoblots with quantification (**B**–**E**) of CTRL and CAL VSMCs transfected with siNC or siHSP90AA1. HSP90AA1 knockdown reduces calcification and reverses Runx2/SM22α expression shifts. (**F**–**J**) Alizarin Red S staining (**F**) and immunoblots with quantification (**G**–**J**) of CTRL and CAL VSMCs transfected with NC or HSP90AA1-overexpressing plasmid. HSP90AA1 overexpression exacerbates calcification and pro-osteogenic phenotypic changes. β-actin, loading control. * *p* < 0.05, ** *p* < 0.01.

To elucidate the functional contribution of HSP90AA1 to VSMC osteogenic transdifferentiation under high calcium/phosphate conditions, we manipulated HSP90AA1 expression in VSMCs using siRNA-mediated knockdown and plasmid-based overexpression. Osteogenic phenotypic changes were assessed by Alizarin Red S staining and Western blot analysis. Alizarin Red S staining revealed that VSMCs cultured in calcification medium developed prominent calcium deposition, whereas HSP90AA1 knockdown significantly attenuated this calcific response (Figure 5A). In line with these observations, Western blot analysis confirmed that siRNA-mediated silencing of HSP90AA1 reduced HSP90AA1 protein levels and was associated with decreased Runx2 and increased SM22α expression in calcification medium-treated VSMCs (Figure 5B–E). We then transfected VSMCs with either the HSP90 overexpression plasmid or an NC plasmid. Alizarin Red S staining demonstrated that VSMCs transfected with the HSP90 overexpression plasmid exhibited exacerbated calcium deposition compared with NC-transfected cells (Figure 5F). Western blot analysis further verified that HSP90 overexpression plasmid transfection significantly increased HSP90AA1 expression in VSMCs and was accompanied by upregulation of Runx2 and downregulation of SM22α under high calcium/phosphate conditions (Figure 5G–J). These data support a modulatory role of HSP90AA1 in VSMC phenotypic switching and calcification, although the downstream mechanism likely requires further clarification.

### 3.6. LAMP2a and HSP90AA1 Co-Localize and Interact in Vascular Calcification

**Figure 6:**
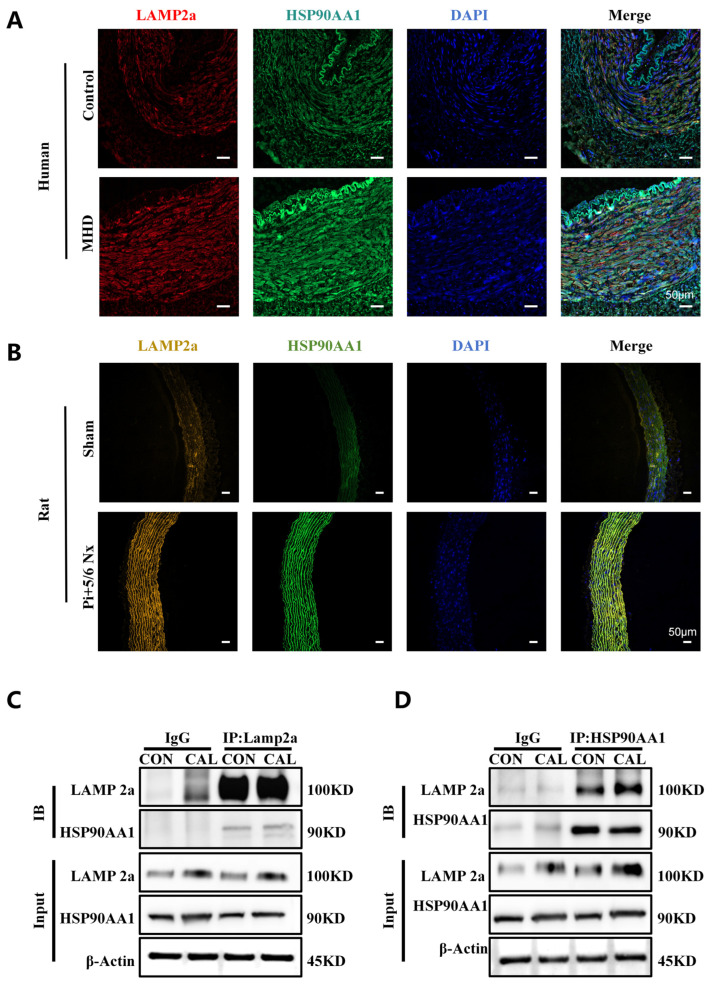
LAMP2a and HSP90AA1 co-localize and interact in vascular calcification. (**A**,**B**) Multiplex immunofluorescence staining shows LAMP2a (red/yellow) and HSP90AA1 (green) co-localization in radial arteries from MHD patients (**A**) and abdominal aortas from Pi + 5/6 Nx rats (**B**), relative to controls. Scale bars: 50 μm. (**C**,**D**) Co-immunoprecipitation assays confirm the physical interaction between LAMP2a and HSP90AA1 in control (CON) and calcified (CAL) VSMCs. β-actin was used as a loading control.

To investigate the spatial relationship between LAMP2a and HSP90AA1 in vivo, we performed multiplex immunofluorescence staining on arterial tissues from MHD patients and CKD rat models. Both LAMP2a and HSP90AA1 were upregulated in calcified tissues compared with controls and showed cytoplasmic co-localization (Figure 6A,B). To further corroborate the interaction between LAMP2a and HSP90AA1, co-immunoprecipitation assays were performed, which confirmed a physical interaction between these two proteins in VSMCs (Figure 6C,D). This co-localization suggests a potential physical interaction between LAMP2a and HSP90AA1 that may underlie their coordinated involvement in regulating VC.

### 3.7. The Regulatory Relationship Between LAMP2a and HSP90AA1

**Figure 7:**
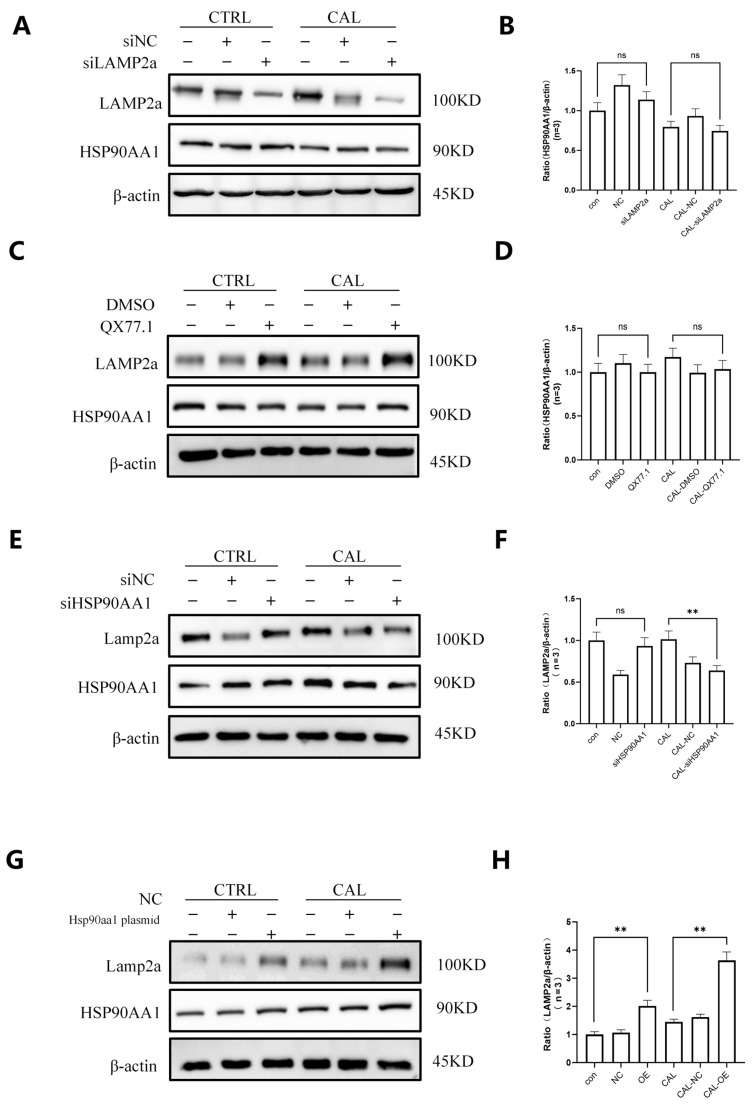
The directional regulatory relationship between HSP90AA1 and LAMP2a. (**A**,**B**) Immunoblots (**A**) and quantification (**B**) show that LAMP2a knockdown does not alter HSP90AA1 expression in control or calcified VSMCs. (**C**,**D**) Immunoblots (**C**) and quantification (**D**) show that CMA activation (QX77.1) does not alter HSP90AA1 expression in CTRL or CAL groups. (**E**,**F**) Immunoblots (**E**) and quantification (**F**) show that HSP90AA1 knockdown (siHSP90AA1) reduces LAMP2a expression in calcified VSMCs. (**G**,**H**) Immunoblots (**G**) and quantification (**H**) show that HSP90AA1 overexpression (OE) increases LAMP2a expression in calcified VSMCs. β-actin was used as a loading control. Data are mean ± SD (*n* = 3). ** *p* < 0.01, ns, not significant.

To further examine the regulatory interplay between LAMP2a and HSP90AA1, we performed respective knockdown and overexpression of LAMP2a and HSP90AA1 in VSMCs. Western blot analysis showed that modulation of LAMP2a did not significantly alter HSP90AA1 expression (Figure 7A–D). In contrast, HSP90AA1 knockdown in VSMCs caused a reduction in LAMP2a expression, while HSP90AA1 overexpression led to an increase in LAMP2a expression (Figure 7E–H). These findings support the possibility that HSP90AA1 acts upstream of LAMP2a, although additional mechanistic studies will be required to define how this regulation occurs.

## 4. Discussion

VC is a serious complication of CKD that substantially increases cardiovascular risk and mortality, particularly in patients undergoing MHD. Central to VC pathogenesis is the osteogenic transdifferentiation of VSMCs, yet the regulatory mechanisms governing this process remain incompletely understood [27,28]. In the present study, we focused specifically on CMA, a selective form of autophagy, and evaluated whether LAMP2a and HSP90AA1 are associated with calcific phenotypic switching in CKD-related VC. Our data provide convergent evidence from human tissues, a CKD rat model, and cultured VSMCs that supports involvement of an HSP90AA1-LAMP2a-CMA pathway in this process.

Our results demonstrate that LAMP2a, the key lysosomal receptor governing CMA, is markedly upregulated in calcified arterial tissues from both MHD patients and CKD rat models (Pi + 5/6 Nx group), accompanied by pronounced alterations in lysosomal morphology, including increased lysosome number and enlarged lysosomal size [29]. These findings are consistent with accumulating evidence implicating autophagy dysregulation in VC [30], although its specific role in CKD-associated calcification has been incompletely understood. In an in vitro model mimicking the uremic milieu of CKD using high calcium and phosphate treatment, we observed that VSMC osteogenic transdifferentiation coincides with enhanced CMA activity, as evidenced by elevated LAMP2a expression and increased lysosomal translocation of PAmCherry1-KFERQ, a CMA-specific substrate. Functional experiments showed that LAMP2a knockdown attenuates calcium deposition, reduces the osteogenic marker RUNX2, and reverses the downregulation of the smooth muscle marker SM22α in calcified VSMCs [31]. In contrast, pharmacological activation of CMA with QX77.1 exacerbated these calcific phenotypes. Taken together, these data support a pivotal role of LAMP2a-dependent CMA in promoting VSMC osteogenic transdifferentiation and VC.

To identify key mediators of CMA in VC, we used a bioinformatics workflow integrating differential gene expression analysis of the GSE146638 dataset, intersection with CMA-associated genes, and PPI network construction [32,33]. This multi-step approach pinpointed HSP90AA1 as a core characteristic gene, exhibiting the highest connectivity within the intersection set and being predicted to interact with LAMP2 via UCHL1. HSP90AA1, a member of the HSP90 chaperone family, is known to regulate protein folding, stability, and intracellular signaling, and has been implicated in autophagy regulation and vascular remodeling [34,35]. We observed that HSP90AA1 was upregulated in calcified arterial tissues and in VSMCs exposed to high calcium/phosphate. Moreover, HSP90AA1 knockdown inhibited VSMCs’ calcification and osteogenic transdifferentiation of VSMCs, whereas overexpression of HSP90AA1 could aggravate VSMCs’ calcification and osteogenic transdifferentiation of VSMCs. These findings support HSP90AA1 as a plausible mediator of calcific phenotypic switching, while also underscoring that the bioinformatic analysis served to nominate candidates for validation [36].

An important aspect of this study is the finding about the relationship between HSP90AA1 and LAMP2a in CKD-associated VC. Multiplex immunofluorescence staining revealed clear co-localization of LAMP2a and HSP90AA1 within the cytoplasm of vascular cells in arterial tissues from MHD patients and CKD rats, providing spatial evidence that these proteins may act in concert in vivo [37,38]. Co-immunoprecipitation further confirmed a physical interaction between LAMP2a and HSP90AA1 in VSMCs, supporting the existence of a protein–protein association relevant to VSMC phenotypic switching. Importantly, mechanistic perturbation experiments suggested a directional regulatory relationship: modulation of LAMP2a expression (knockdown or overexpression) did not significantly alter HSP90AA1 levels, whereas HSP90AA1 knockdown decreased LAMP2a expression and HSP90AA1 overexpression increased LAMP2a expression. Together, these findings support a model in which HSP90AA1 functions upstream to positively regulate LAMP2a-associated CMA activity, thereby promoting VSMC osteogenic transdifferentiation and vascular calcification. Nevertheless, the current data do not fully define whether HSP90AA1 regulates LAMP2a stability, trafficking, assembly into CMA complexes, or another upstream step. Therefore, the proposed HSP90AA1-LAMP2a-CMA axis should be regarded as a working mechanistic model supported by the present data rather than as a fully established pathway.

Several limitations should be acknowledged. First, the analysis results of human arterial vascular tissues were based on a small sample set. Second, although the vascular tissues of the non-CKD control group were obtained from patients without underlying diseases who required surgical amputation due to acute trauma, they may still not fully represent a healthy vascular control group. Third, the animal model included two experimental groups and did not incorporate additional sham/high-phosphate or nephrectomy/normal-diet comparator groups. Fourth, although the 5/6 nephrectomy plus high-phosphate diet model was already a relatively classic CKD animal model and reproduced important features of CKD-related VC, it could not fully recapitulate the complex uremic milieu in humans. Fifth, although we observed functional links between HSP90AA1, LAMP2a, and calcification, the precise molecular mechanism by which HSP90AA1 regulates CMA remains to be fully elucidated [39]. Finally, potential crosstalk between CMA and other autophagy pathways [13], including macroautophagy, was not addressed in this study.

Despite these limitations, the study adds to the emerging literature indicating that CMA pathways may participate in vascular calcification with important potential clinical implications. The coordinated upregulation of LAMP2a and HSP90AA1 in calcified tissues, their direct physical interaction, and their synergistic promotion of VSMC osteogenic transdifferentiation collectively highlight these molecules as promising therapeutic targets in CKD-related VC [40]. Pharmacological or genetic strategies aimed at modulating CMA may therefore represent a viable approach to limit or reverse VC progression [41]. Future work should focus on developing specific modulators of this pathway and rigorously evaluating their efficacy and safety in preclinical and clinical settings. In addition, exploring the potential of LAMP2a and HSP90AA1 as diagnostic or prognostic biomarkers might provide clinicians with useful tools for risk stratification and individualized management of CKD patients at high risk for VC [42,43], although this possibility will require validation in larger cohorts and in more rigorous mechanistic and translational studies.

In summary, the present study supports an association between increased CMA activity and CKD-related vascular calcification and identifies HSP90AA1 as a candidate upstream regulator linked to LAMP2a expression. These findings refine current understanding of CKD-related VC pathogenesis and provide a basis for future mechanistic and translational studies of the HSP90AA1-LAMP2a-CMA pathway.

## Data Availability

The data supporting this study are available from the corresponding author upon request.

## Supplementary Materials

The following supporting information can be downloaded at: https://www.mdpi.com/article/10.3390/biomedicines14040881/s1, Figure S1: Alizarin Red S staining of VSMCs following calcification induction with 10 mM β-glycerophosphate and 1.5 mM calcium for different durations.

## Abbreviations

The following abbreviations are used in this manuscript:

CKD	Chronic kidney disease
VC	vascular calcification
VSMCs	vascular smooth muscle cells
CMA	Chaperone-mediated autophagy
LAMP2a	lysosome-associated membrane protein 2A
ESRD	End-Stage Renal Disease
MHD	Maintenance hemodialysis
PPI	Protein–protein interaction
Co-IP	Co-immunoprecipitation
IHC	Immunohistochemistry
IF	Immunofluorescence
TEM	Transmission electron microscopy
DEGs	Differentially expressed genes
GEO	Gene Expression Omnibus
