# Supplementary figures and images for: HSP90AA1 Facilitates Vascular Calcification in Chronic Kidney Disease Involving Chaperone-Mediated Autophagy
Source: Biomedicines. 2026 Apr 12;14(4):881. doi: 10.3390/biomedicines14040881 (PMC13113582; doi:10.3390/biomedicines14040881)

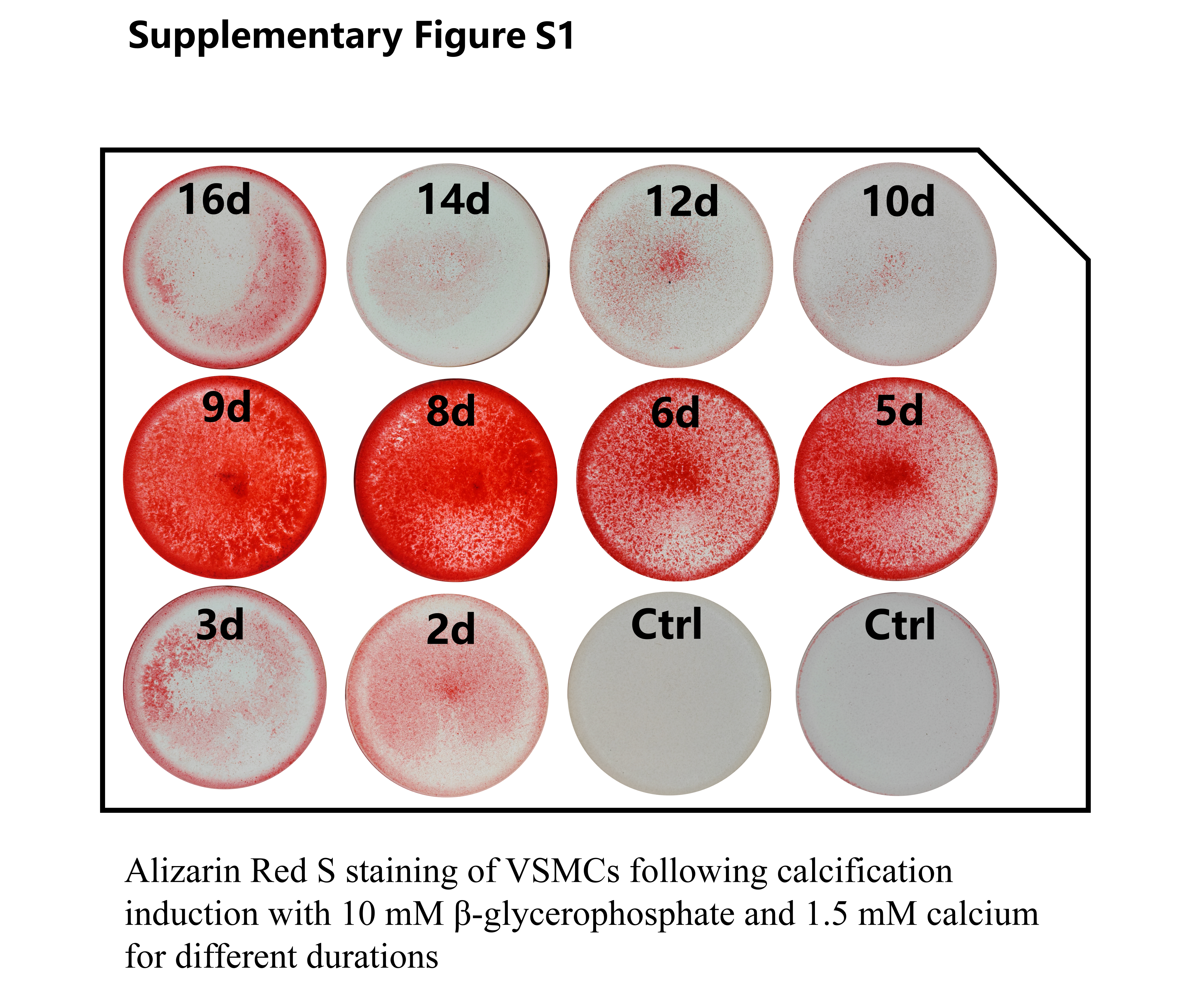

Supplement: Supplementary file 1 [file biomedicines-14-00881-s001.zip › Supplementary Figure S1.tif]
